# QTL Mapping of Stem Rust Resistance in Populations of Durum Wheat

**DOI:** 10.3390/genes13101793

**Published:** 2022-10-04

**Authors:** Daniela Marone, Elisabetta Mazzucotelli, Oadi Matny, Francesca Desiderio, Giuseppe Sciara, Marco Maccaferri, Ilaria Marcotuli, Agata Gadaleta, Brian Steffenson, Anna Maria Mastrangelo

**Affiliations:** 1 Council for Agricultural Research and Economics, Research Centre for Cereal and Industrial Crops, 71122 Foggia, Italy; 2 Council for Agricultural Research and Economics, Research Centre for Genomics and Bioinfomatics, 29017 Fiorenzuola d’Arda, Italy; 3 Department of Plant Pathology, University of Minnesota, Saint Paul, MN 55108, USA; 4 Department of Agricultural Sciences, University of Bologna, 40127 Bologna, Italy; 5 Research Unit of “Genetics and Plant Biotechnology”, Department of Agricultural & Environmental Science, University of Bari, 70126 Bari, Italy

**Keywords:** tetraploid wheat, stem rust, resistance loci

## Abstract

Stem rinfectionust, caused by the fungus *Puccinia graminis* f. sp. *tritici* (*Pgt*), is one of the most devastating fungal diseases of durum and common wheat worldwide. The identification of sources of resistance and the validation of QTLs identified through genome-wide association studies is of paramount importance for reducing the losses caused by this disease to wheat grain yield and quality. Four segregating populations whose parents showed contrasting reactions to some *Pgt* races were assessed in the present study, and 14 QTLs were identified on chromosomes 3A, 4A, 6A, and 6B, with some regions in common between different segregating populations. Several QTLs were mapped to chromosomal regions coincident with previously mapped stem rust resistance loci; however, their reaction to different *Pgt* races suggest that novel genes or alleles could be present on chromosomes 3A and 6B. Putative candidate genes with a disease-related functional annotation have been identified in the QTL regions based on information available from the reference genome of durum cv. ‘Svevo’.

## 1. Introduction

Durum wheat (*Triticum turgidum* L. var. *durum*) is a tetraploid wheat species mainly used for the production of couscous and alimentary pasta. In Mediterranean countries, it is economically important as it accounts for approximately 75% of global durum wheat production [1]. Stem rust is one of the most devastating fungal diseases for durum and common wheat worldwide. It is caused by the fungus *Puccinia graminis* f. sp. *tritici* (*Pgt*). New widely virulent races of *Pgt* occasionally emerge and spread, threatening wheat production on a global scale. For example, in the United States races TPMKC and TTTTF overcame several widely used Sr genes [2,3]; in Africa, race TTKSK (isolate Ug99) was first discovered in Uganda and was found to carry virulence for the widely deployed resistance gene *Sr31*. Variants in the Ug99 lineage possess virulence for *Sr24*, *Sr36*, *Sr9h*, and *SrTmp* and have spread across the eastern and southern part of the continent [4,5,6]. In Ethiopia, three races of *Pgt* were described, namely TRTTF, JRCQC, and TKTTF, with virulence on resistance genes *Sr9e* and *SrTmp* respectively [7,8]. Isolated stem rust outbreaks have also occurred in recent years in regions where the disease was largely eradicated in the mid-to-late twentieth century, e.g., in Western Europe. Lewis et al. [9] reported the first wheat stem rust occurrence in the United Kingdom in nearly 60 years, with only 20% of U.K. wheat varieties showing resistance to the strain (UK-01) causing the outbreak. In the same year, *Pgt* race PTKSK, virulent on many currently used resistance genes, was reported for the first time in South Africa [10]. These reports are due to the emergence of new virulent races and are likely favored by ongoing climate changes and the pattern of long-range atmospheric transport, which is an important mode of spread for *Pgt*. By applying prediction models, Prank et al. [11] suggested that warmer climates with lower relative humidity and enhanced turbulence can increase urediniospore dispersal from infected fields leading to disease spread on a global scale. At the same time, the overwintering areas of the fungus are predicted to expand due to a reduction in subfreezing conditions. Under these scenarios, the identification of new sources of genetic resistance is the best strategy to reduce the impact of stem rust on wheat production in the frame of sustainable agricultural systems and banning of chemical phytoprotection. In particular, genes conferring broader and more durable resistance or combinations of resistance genes are needed in ongoing breeding programs. More than sixty *Sr* genes have been identified and genetically mapped in wheat [12,13]. However, most of these provide complete resistance against specific *Pgt* races but can be overcome by other races of the pathogen based on a gene-for-gene model. Many race-specific stem rust resistance genes have been cloned, including *Sr35* [14], *Sr33* [15], *Sr50* [16], *Sr22* and *Sr45* [17], *Sr13* [18], *Sr21* [19], *Sr46* [20], and *Sr60* [21]. These genes all encode coiled-coil nucleotide-binding leucine-rich repeat (NLR) proteins.

Slow-rusting genes often confer uniform resistance to many rust pathogen races as well as against different pathogen species such as powdery mildew, Barley Yellow Dwarf Virus, and spot blotch. These include *Sr2/Yr30*, *Sr55/Lr67/Yr46/Pm46*, *Sr57/Lr34/Yr18/Pm38/Sb1/Bdv1*, and *Sr58/Lr46/Yr29/Pm39* [22,23,24,25]. Two of of the genes in these complexes have been cloned: *Sr57/Lr34* and *Sr55/Lr67*, which encode a putative ABC transporter [26] and a hexose transporter [27], respectively.

Studies of the genetic basis of stem rust resistance have also revealed the role of resistance suppressor genes such as *SuSr-D1*, a gene identified in the bread wheat cultivar ‘Canthatch’ that can suppress resistance to *Pgt* and whose mutations confer resistance to several races of *Pgt* which are instead virulent on wild-type plants [28]. Moreover, mutations leading to non-functional pathogen avirulence genes *AvrSr50* and *AvrSr35* have been associated with the appearance of virulence in *Pgt* races towards plants carrying *Sr50* and *Sr35*, respectively [29,30]. Therefore, studying cognate genes in the pathogen adds another layer of knowledge on pathogenicity and resistance mechanisms.

*Sr* genes have a different origin and some have been identified in wild relatives. As an example, *Sr35* and *Sr60* were derived from *T. monococcum* [14,31], while *Sr33* [15] and *Sr45* [17] were from *Aegilops tauschii* Coss. Once identified, these genes can be introgressed into bread or durum wheat genetic backgrounds through pre-breeding schemes. Identifying resistance genes directly in cultivars offers the advantage of having the gene already available in an adapted genetic background, thereby facilitating its rapid and easy transfer to other elite cultivars without the problem of linkage drag.

Screening durum wheat germplasm is necessary to identify new sources of resistance, and large panels have been developed and made available to the scientific community together with genotypic single nucleotide polymorphism (SNP) data [32,33]. Genome-wide association studies (GWAS) have been used to identify and map genes and QTLs for resistance to *Pgt* in tetraploid wheat [34,35], together with QTL mapping in biparental recombinant inbred line (RIL) populations [36,37]. The combined use of different populations allows one to identify more resistance loci and provides strong evidence of their effect, when validated in different populations. Moreover, multi-parental cross designs for mapping QTL are characterized by a broader genetic basis and potentially higher mapping resolution [38].

A GWAS approach was previously applied to a panel of tetraploid wheat genotypes to identify QTLs for resistance to different races of *Pgt*. This indicated that some resistance genes were carried by durum wheat genotypes which are parents of established RIL populations [34,35]. The aim of the present study was therefore to carry out a careful phenotypic analysis of the reaction of such RIL populations, in particular, three biparental and one multi-parental RIL population, in order to identify the genetic determinants involved in stem rust resistance in a tetraploid background. The confidence intervals of the QTLs were determined to identify chromosomal regions involved in *Pgt* resistance in common between different mapping populations. Moreover, a search of candidate genes was carried out by projecting the identified QTLs onto the genome sequence of the reference durum wheat cv. ‘Svevo’.

## 2. Results

### 2.1. Evaluation of Four Segregating Populations with Their Parents for Reaction to Stem Rust

In a first phenotypic evaluation, the infection types (ITs) of the 9 parental genotypes were assessed with the following *Pgt* races under controlled conditions: TTTTF, TPMKC, JRCQC, and TKTTF. The aim of this experiment was to identify the races suitable for mapping resistance loci in the available segregating populations. The parents of the ‘Latino’ × ‘MG5323’ (LatMG) RIL population showed a contrasting reaction to three races. In particular, *T. dicoccum* accession ‘MG5323’ showed a high level of resistance to *Pgt* races TPMKC, TKTTF and JRCQC (ITs = 1, 0.3, and 0.2, respectively), while cultivar ‘Latino’ was susceptible (ITs = 8.5, 9 and 8.2, respectively). Two races were discriminant for the RIL population ‘Ciccio’ × ‘Svevo’ (CicSve): cultivar ‘Ciccio’ was susceptible to races TPMKC and TKTTF (IT = 9 in both cases), while ‘Svevo’ showed moderate resistance (ITs = 2 and 3.7, respectively); the two cultivars were instead both resistant to TTTTF and susceptible to JRCQC. For the ‘Cirillo’ × ‘Neodur’ (CirNeo) population, both of the parents showed a resistant reaction against race TTTTF but exhibited distinctly different ITs (ITs = 1.4 and 0, respectively), suggesting the presence of two different resistance genes in the two genotypes. ‘Neodur’ was also one of the four parents of the NCCR multi-parent population, together with ‘Claudio’, ‘Colosseo’ and ‘Rascon/2*Tarro’. Two of these latter three parents showed resistant ITs to TTTTF (0.3 for Claudio and 1.7 for ‘Rascon/2*Tarro’), the exception being ‘Colosseo’, which exhibited a susceptible reaction (IT = 8.3). Based on these results, the segregating populations LatMG, CicSve, CirNeo, and NCCR were investigated for rust reaction to the corresponding discriminating race of the parents at the seedling stage under controlled conditions. In addition, the NCCR multi-parent mapping population was expected to increase the mapping resolution of the resistance loci carried by the three resistant parents. In the different experiments, high infection levels and ITs were observed on the susceptible checks, demonstrating optimal infection levels for the clear scoring of rust reactions in the segregating populations.

**Figure 1:**
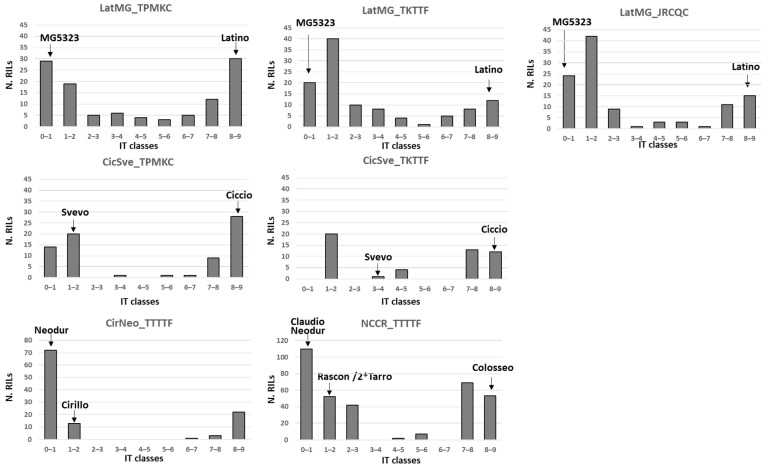
Frequency distribution of the ITs in the four segregating populations. The phenotypic class for each parent is indicated by the arrow.

The CicSve population had low germination rates; however, it was considered for possible validation of genes segregating in other populations. As a first step, the frequency distribution of phenotypic reactions for the four RIL populations was analyzed to understand the genetic complexity of rust resistance in the different populations (Figure 1). In all cases, the frequency distribution of IT scoring was bimodal with a majority of highly resistant and highly susceptible lines, and only few lines displaying intermediate IT values. This profile suggests resistance being based on one or a few genes segregating within each population.

A chi-square test was applied to compare the observed segregation ratios with the ones expected upon segregation of one or a few major genes (Table 1). As suggested by a similar number of RILs in the resistant and susceptible classes, one major resistance gene was predicted to segregate in the LatMG and CicSve populations in response to TPKMC and in the CicSve population in response to TKTTF. The number of resistant lines was significantly higher than the number of susceptible ones in the CirNeo population to TTTTF and in the LatMG population to TKTTF and JRCQC, suggesting that two independent resistance genes controlled the phenotypic response. Lastly, the bimodal frequency distribution observed for the NCCR with an estimated ratio of 5 (resistant): 3 (susceptible) suggested a more complex genetic model. Claudio and Neodur may share the same resistance gene since they showed the same IT in response to the race TTTTF.

An ANOVA was carried out to assess genotype differences in the four segregating populations. Statistically significant differences (*p <* 0.001) were found across genotypes for all the populations and the *Pgt* races used in the study. A very good repeatability was observed for this experiment, as indicated by the heritability values, ranging from 73% for race TKTTF in CicSve population to 99% for the NCCR population tested with the race TTTTF, highlighting the robustness of the data and the low error rate (Table 2).

Correlation analysis was also conducted for the reaction of RILs belonging to the same mapping population against the different races. Significant correlations were observed for all contrasts evaluated (Table 3), with higher values found in response to races TKTTF and TPKMC in the CicSve population and to races TKTTF and JRCQC in the LatMg population.

### 2.2. QTL and Haplotype Analysis

**Figure 2:**
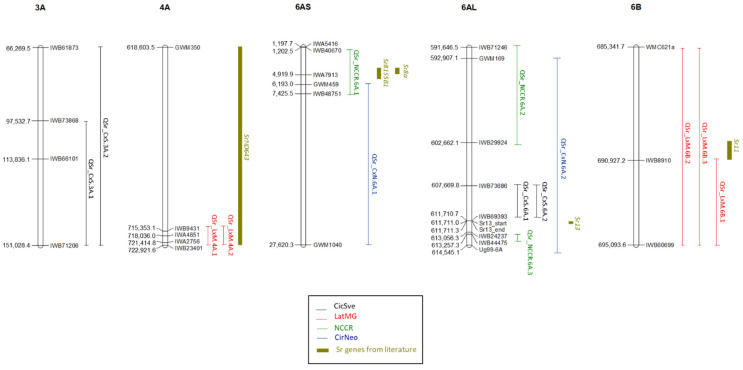
Physical map position of the QTLs identified in the present study and of the known resistance genes mapped to the same regions.

A total of 14 QTLs were identified considering all the populations and the *Pgt* races assessed (Table 4, Figure 2). The most important QTLs in terms of LOD score and percentage of explained phenotypic variation were located on chromosomes 6A and 6B. In further detail, two distinct regions were found on chromosome 6A. The first one, in the distal portion of the short arm, was identified in response to race TTTTF in the RIL population CirNeo, QSr_CxN.6A.1, and in the multi-parental population NCCR, QSr_NCCR.6A.1. Interestingly, based on the additive effects, the resistant allele underlying this QTL was carried by Neodur in both populations. The LOD value was 13.27 in CirNeo and 39.1 in NCCR with a percentage of observed phenotypic variation of 42% and 30% in the two populations, respectively. The confidence interval was much larger for CirNeo compared to NCCR population, due to the low number of molecular markers present in the corresponding genetic map, and it overlapped with QSr_NCCR.6A.1 in the region comprised between 6.2 and 7.4 Mb. Supplementary Table S1 reports the allelic state of SNP markers underlying QTLs on chromosome 6A for the parents of the segregating populations and a group of durum cultivars which carry different resistant haplotypes at *Sr13* locus (‘Kronos’, ‘Altar 84′, ‘Langdon’, ‘Lloyd’, and ‘Medora’). Based on genotypic analysis, this is a region characterized by an extended polymorphism between ‘Neodur’ and the other parents of the NCCR population on one side, but quite monomorphic between ‘Neodur’ and ‘Cirillo’, except for the marker IWB8452, co-mapping with Xgwm459, the peak marker of QSr_CxN.6A.1 at 6.2 Mb (Supplementary Table S1). Interestingly, these results can help in reducing the size of the 6AS region in which the resistance gene is located.

This region corresponds to the chromosome interval where the stem rust resistance genes *Sr8a* was previously mapped (Figure 2).

A second region conferring resistance to TTTTF was identified on the long arm of chromosome 6A. Similar to what was seen for the short arm, a QTL with a large confidence interval was found in CirNeo, QSr_CxN.6A.2, and meanwhile two distinct QTLs were detected in NCCR: QSr_NCCR.6A.2 and QSr_NCCR.6A.3. Based on the sign of the additive effect for QSr_CxN.6A.2 on chromosome 6AL, the resistance allele was contributed by ‘Cirillo’, whereas for QSr_NCCR.6A.2 and QSr_NCCR.6A.3, mapped in the NCCR population, the resistant haplotype was carried by ‘Claudio’ and ‘Rascon/2*Tarro’. For QSr_NCCR.6A.2, a very large haplotype (57 SNPs) except for just one marker (IWB16528), was shared by the two resistant parents which were different with respect to the susceptible parents, ‘Neodur’ and ‘Colosseo’ (Supplementary Table S1).

In a very close position, two more QTLs, QSr_CxS.6A.1 and QSr_CxS.6A.2, were mapped in the RIL population CicSve, but for resistance to different races, TKTTF and TPKMC, respectively. In both cases, the resistance allele was contributed by ‘Svevo’. The physical position of the resistance gene *Sr13* (611.8 Mb) is at the position of the peak marker of QTLs QSr_CxS.6A.1 and QSr_CxS.6A.2 (IWB69393) and within the confidence interval of QSr_CxN.6A.2, while QSr_NCCR.6A.3 is located less than 2 Mb distal to *Sr13* position, at 613.2 Mb. For haplotypes in the region surrounding *Sr13*, a general monomorphism is observed across the considered genotypes, except for ‘Colosseo’, but the SNP corresponding to *Sr13*, IWB69393, is polymorphic between ‘Ciccio’ and ‘Svevo’, ‘Cirillo’ and ‘Neodur’, and between ‘Claudio’ and ‘Rascon/2*Tarro’ on one side and ‘Colosseo’ and ‘Neodur’ on the other side. Interestingly, the BB allelic state is shown by all the resistant parents of the segregating populations considered in the present study.

Considering the three populations, LOD values ranged from 9.9 in CirNeo to race TTTTF to 70.9 in NCCR to the same race, with percentage of observed phenotypic variation ranging from 34% in CirNeo to 92% in CicSve for race TPMKC. QTL QSr_NCCR.6A.2 showed a LOD value of 54.9 with 36% observed phenotypic variation.

Other chromosomal regions with high LOD values were identified on chromosomes 4A and 6B in the RIL population LatMG. In the first region, two coincident QTLs, QSr_LxM.4A.1 and QSr_LxM.4A.2, were mapped to the long arm of chromosome 4A in response to JRCQC and TKTTF, while the QTL on chromosome 6B explained resistance to the three *Pgt* races: TPKMC, JRCQC and TKTTF. For all these regions, the *T. dicoccum* accession MG5323 contributed the alleles for resistance. The QTL on chromosome 6B mapped to a position coincident with *Sr11* (Figure 2).

Overall, the expected number of genes predicted based on the frequency distribution for rust reaction (Figure 1) and the χ^2^ test were confirmed for the populations CirNeo and LatMG.

### 2.3. Identification of Candidate Genes in the QTL Intervals

The search for candidate genes was carried out considering the genes present in the confidence interval of the QTLs identified in the present study. The SNP markers included in the QTL intervals were projected onto the reference genome sequence of durum wheat (cv. ‘Svevo’ [32]), and the functional annotation of the genes included within the corresponding interval of the physical map was inspected (Table 5 and Table S2). The physical confidence intervals of the QTLs ranged between 0.2 and 84.8 Mbp, and the number of annotated genes retrieved in these regions ranged from 18 for QTL QSr_NCCR.6A.3 to 1136 for QTL QSr_CxN.6A.2.

The number of annotated genes was not strictly related to the size interval in Mbp; indeed, the ratio between the number of annotated genes and interval size was highest for QTL QSr_NCCR.6A.3 with 89.5 genes per Mbp. A functional annotation was available for many of these genes, so it was possible to identify those involved with a disease response. This class of genes included those annotated as very similar to known genes conferring resistant or susceptible reactions in several plant species or genes belonging to gene families known to have a role in plant immunity, such as nucleotide-binding and leucine-rich repeat immune receptor (*NLRs*) genes. Several of the most important identified regions harbored a high number of disease-response genes, probably organized in gene clusters derived from tandem duplication events. Twenty disease-related genes were identified from a total of 139 genes in the region of QSr_LxM.4A.1/2. Within this region, genes annotated as *NLRs* formed a cluster of six genes very close to each other at 721 Mb. *NLR* genes and LRR receptor-like serine threonine-kinases were also present in this QTL region around the position of the peak marker (725.7–726.5 Mb). A very large cluster of *NLR* genes, genes annotated as *RPM1* (*Resistance to Pseudomonas syringae pv. Maculicola 1*), and *TIR-NBS-LRR* genes are present in the region of QSr_LxM.6B.2 and 3, and a gene corresponding to *RPM1* is present at the peak of the QTL (695 Mb). For QTLs located on the long arm of chromosome 6A, a cluster of eight genes annotated as coding for receptor protein kinases and one RPM1 are present at the left side of the QTL, near the peak marker in the region of QSr_NCCR.6A.2. For QTLs QSr_CxN.6A.2, QSr_CxS.6A.1 and QSr_CxS.6A.2, a gene annotated as disease-resistant *RPM1* is present at the position of *Sr13* (611.7 Mb).

Besides genes conferring specific reactions to pathogens, other genes involved in general responses to pathogen attack were also identified, such as those annotated as callose synthase, observed in QSr_CxN.6A.1, polygalacturonase in 3A_QSr_CxS.3A.1 and 3A_QSr_CxS.3A.2, and those related to flavonoid biosynthesis, which were observed in both QSr_CxN.6A.1 and QSr_NCCR.6A.1.

## 3. Discussion

Stem rust and other fungal diseases are responsible for significant losses to wheat grain yield and quality worldwide. Moreover, as new virulent pathogen races continue to emerge within the context of climate change, greater and more widespread production losses are possible. Enhancing genetic resistance to *Pgt* is a powerful and sustainable alternative to the widespread use of fungicides for rust mitigation but requires the continuous identification and characterization of resistance genes in wheat gene pools.

Based on recent studies carried out on tetraploid wheat RIL populations and accession panels through GWAS, stem rust resistance is likely under oligogenic or polygenic additive control, with multiple loci and beneficial alleles of variable effect (major and minor) acting in a cumulative way [32,39,40]. Currently, many tetraploid wheat accessions are available for exploration of genetic and phenotypic variation with respect to stem rust resistance [32,33]. Wild and domesticated emmer accessions are a valuable source of resistance alleles which can be easily transferred into durum and also to common wheat cultivars [41]. On the other hand, identifying useful alleles for stem rust resistance in elite durum cultivars represents a great advantage in breeding as the resistance determinants are already present in an adapted genetic background with respect to agronomic and quality traits and ready for cultivation in regions in which pedo-climatic conditions are more favorable to *Pgt* development. Moreover, the transfer of a resistance locus from a wild accession runs the risk of introducing linked deleterious alleles (i.e., linkage drag) in the recurrent parent. For these reasons, a genetic analysis was carried out in the present study on RIL populations derived from elite durum cultivars and a domesticated emmer accession belonging to tetraploid wheat panels previously characterized for stem rust resistance at the seedling stage [34,35,40,42].

The parents of the RIL populations showed contrasting reactions to races of *Pgt*, except in the case of ‘Cirillo’ and ‘Neodur’, where both were resistant to race TTTTF but with different ITs. Postulation of resistance genes in the parental lines was attempted by comparing their ITs to the five *Pgt* races with those exhibited by the 20 wheat stem rust differential lines. A correspondence between individual parents and differential lines was found only for ‘Latino’ and ‘MG5323′. In particular, ‘Latino’ showed a resistance spectrum similar to a number of differential lines, including those carrying several alleles at the *Sr9* locus, possibly *Sr9e*. ‘MG5323′ showed a resistance spectrum similar to lines carrying *Sr24* (Supplementary Table S3), but the map position of this gene on chromosome 3D is not coincident with the QTLs identified in the LatMG population; therefore, we could not draw clear conclusions from the analysis of the differential lines. In all populations, the frequency distribution of the linearized IT values suggested the presence of one or a few genes explaining the resistant phenotype, and these indications were confirmed by the results of the QTL analysis. A total of 14 QTLs were identified in the different populations in response to the *Pgt* races used in the present study. Some of these QTLs were of particular interest because they conferred resistance to the same *Pgt* race but in different populations. Such was the case for the QTLs identified on chromosome 6A. The QTLs QSr_CxN.6A.1 and QSr_NCCR.6A.1 were mapped to the telomeric region of the short arm of chromosome 6A for reaction to race TTTTF. In both populations, the resistance gene was carried by cultivar ‘Neodur’. Inspection of haplotypic state of the parents of the segregating populations herein analyzed strongly suggests that the region comprised between 6.2 and 7.4 Mb may contain the resistance gene, very close to markers IWB8452 and Xgwm459 at 6.2 Mb (Supplementary Table S1). For resistance to stem rust, QTLs and MTAs were previously mapped in this region, but not to the same *Pgt* race. MTAs were found at 4.2 Mb in a panel of 183 durum wheat lines following natural infection in the field [40] and between 0.5 and 4.9 Mb to a mix of different *Pgt* races, including TTTTF [43]. Saini et al. [44] found a QTL in the same region in a segregating population derived from a cross between ‘Lebsock’ and the *T. turgidum* ssp. *carthlicum* accession PI 94749, but the QTL was for a reaction to race TRTTF. The authors also considered the race TTTTF, for which no QTL was identified on the short arm of chromosome 6A. Babiker et al. [37] investigated a bread wheat population and identified a QTL for resistance to race TTTTF on chromosome 6DS and also a QTL on 6AS, but only in response to race TRTTF. The resistance genes *Sr8* and *Sr8155B1* were previously mapped in this region. *Sr8a*, carried by the Canadian spring wheat cv. ‘Harvest’, is effective against TRTTF according to the profile of the QTLs previously mapped and described above [36]. *Sr8155B1*, mapped in durum wheat, is effective against TTKST and TRTTF [45]. In particular, *Sr8155B1*, considered a new allele of *Sr8a*, has been located between 6.7 and 10.9 Mb [46], and therefore may correspond to QSr_NCCR.6A.1. Collectively, these data suggest that the QTL identified in our populations could be very close but different with respect to those already mapped, or possibly a new allele with a different race specificity. Side by side evaluations of all these resistance sources with parents of the mapping populations analyzed in the present study could be helpful perspective to closely examine the reactions to see if they are similar and therefore containing the same genes.

The second region of interest on chromosome 6A was found on the long arm in the CirNeo and NCCR populations in response to race TTTTF and in the CicSve population in response to races TKTTF and TPMKC. The parents carrying the resistance allele in this region include ‘Cirillo’, ‘Claudio’, ‘Rascon/2*Tarro’, and ‘Svevo’. Interestingly, the large number of lines and the four different parents of the NCCR population allowed the increase in the resolution in the genetic analysis, and to detect two distinct QTLs instead of the unique QTL identified in the CirNeo population. QTL QSr_NCCR.6A.2, mapped between 591.6 and 602.7 Mb may correspond to the one located at 598.6 Mb by [43]. Due to the small number of markers in the CirNeo genetic map, a larger confidence interval was observed for QSr_CxN.6A.2. This region is important because it contains critical stem rust resistance loci effective against several *Pgt* races, including TTKSK and TTTTF [34,35,40,42,47]. The same QTL conferring resistance to race TTKSK was identified in the CirNeo population with ‘Cirillo’ being the donor [34]. Moreover, in this region, *Sr13* was genetically mapped [47] and physically positioned at 611.8 Mb [18]. The gene lies at the distal end (position of the peak marker IWB69393) of the confidence interval of QTLs QSr_CxS.6A.1 and QSr_CxS.6A.2 and within the confidence interval of QSr_CxN.6A.2, while QSr_NCCR.6A.3 is located less than 2 Mb distal to *Sr13* position, at 613.2 Mb. Interestingly, the SNP corresponding to *Sr13*, IWB69393, is polymorphic between ‘Ciccio’ and ‘Svevo’, ‘Cirillo’, and ‘Neodur’, and between ‘Claudio’ and ‘Rascon/2*Tarro’ on one side and ‘Colosseo’ and ‘Neodur’ on the other one, despite a general monomorphism is observed around this marker (Supplementary Table S1). Moreover, all the resistant parents of the segregating populations considered in the present study share the BB allelic state, together with the cultivars ‘Kronos’, ‘Altar 84′, ‘Langdon’, ‘Lloyd’, and ‘Medora’, which carry different resistant haplotypes at *Sr13* locus, in detail ‘Kronos’ carries *Sr13a*, ‘Svevo’, ‘Lloyd’ and ‘Medora’ *Sr13b*, ‘Altar 84′ and ‘Langdon’ *Sr13c* [48]. *Sr13b* was shown to confer resistance to TKTTF [18], to TPMKC and to TTTTF [48], but CicSve population was not tested against this race since the two parents were both resistant, although at a different level. The fact that the confidence interval of QTL QSr_NCCR.6A.3 does not include *Sr13* suggests two possible explanations. It could represent a different and novel *Pgt* resistance locus, or, more probable hypothesis, it also corresponds to *Sr13*. Indeed, in the region at 613 Mb in which the QTL has been detected, ‘Claudio’ and ‘Rascon/2*Tarro’ show the same allele at the markers mapped to this location, which are in turn different with respect to the allele shared by ‘Neodur’ and ‘Colosseo’. On the contrary, for the markers mapped around *Sr13* in the NCCR genetic map, ‘Claudio’ and ‘Rascon/2*Tarro’ share the same alleles as ‘Neodur’, therefore the effect is masked and the LOD value does not reach the significant threshold in this region. The marker IWB69393, corresponding to *Sr13*, is indeed polymorphic between resistant and susceptible parents of the NCCR population, but it has been discarded from the analysis for the high rate of missing data.

QTLs QSr_CxS.3A.1 and QSr_CxS.3A.2 conferring resistance to races TKTTF and TPMKC, respectively, were mapped to chromosome 3A and are very close to an MTA involved in resistance to *Pgt* races TTTTF and JRCQC [42] (146.5 Mb). QTLs QSr_LxM.4A.1 and QSr_LxM.4A.2, conferring resistance to races JRCQC and TKTTF, respectively, were mapped to chromosome 4A very close to MTAs for resistance to the same races, TKTTF and JRCQC [35], and field populations of the pathogen identified in a collection of durum wheat lines [40,42]. Indeed, the MTAs identified by Saccomanno et al. [35] were positioned at 719 and 734 Mb, within the interval of QSr_LxM.4A.1 and QSr_LxM.4A.2. The same was true for MTAs mapped by Letta et al. [40,42] positioned at 723.9 and 719.4 Mb, respectively.

The QTLs identified in the LatMG population in response to *Pgt* races TPMKC, JRCQC, and TKTTF on chromosome 6B were very close to an MTA identified at 692.2 Mb by Megerssa et al. [43] in a panel of durum wheat lines following infection in the field with a composite of different races including those tested in the present study. In the same region Nirmala et al. [49] mapped *Sr11*, which is effective against TKTTF and ineffective against TTKSK, JRCQC, and TRTTF. These results suggest that more than one gene could be carried by ‘MG5323’ in this region or that ‘MG5323’ carries an allele with a different resistance spectrum.

The availability of a reference genome sequence for crops is a valuable asset for investigating the gene content of identified QTL intervals and to identify candidate genes for cloning once the above-mentioned interval is reduced to a very small region by fine mapping. The gene content of QTLs identified in the present study was investigated by projecting the sequence of the corresponding SNP markers within the QTL confidence interval to the genome of durum wheat cultivar ‘Svevo’ [32]. In general, smaller intervals in Mb were found for the LatMg and NCCR populations, the first characterized by a domesticated emmer parent and the second by a four-way cross for which a higher resolution was expected. The number of the genes residing in these intervals is very high, usually in the hundreds. Their annotation has been inspected to identify genes potentially involved in disease resistance. Gene families implicated in disease response often contain many members, for example in the case of *NLR* type genes [50,51]. Thus, the probability of identifying one of these genes in a given interval by chance is high. Nevertheless, finding clusters of such genes at the position of the peak marker or in a very close position can help in identifying interesting candidate genes for further research. This is the case for QSr_LxM.4A.1, for which a cluster of six genes annotated as NBS-LRR class disease resistance, lies right at the position of the QTL peak (725.7–726.5 Mb). Similarly, two genes annotated as protein kinase are present in the region between 6.7 and 7.4 Mb, where QTL QSr_CxN.6A.1 and QSr_NCCR.6A.1 overlap, and where haplotype analysis also suggests the presence of the resistance gene for these QTLs. From this point of view, the presence of such genes in QTLs which may be novel, as the QTLs mapped to the short arm of chromosome 6A, QSr_NCCR.6A.1, and those carried by MG5323 on chromosome 6B, establishes them as the most suitable QTLs for validation and fine mapping. In the meantime, a set of durum wheat cultivars characterized for resistance to one or two *Pgt* races have been described, which are not only highly valuable in current durum production but are also useful genetic resources for future durum breeding.

## 4. Materials and Methods

### 4.1. Plant Materials and Races of the Stem Rust Pathogen

Four segregating RIL populations were evaluated for disease reaction to races of *Pgt*. These included two biparental populations made between durum wheat cultivars (‘Ciccio’ × ‘Svevo’ (CicSve) with number of RILs 120 [52] and ‘Cirillo’ × ‘Neodur’ (CirNeo) with 148 RILs) [53]; one biparental population obtained by crossing the durum wheat cultivar ‘Latino’ to the *T. dicoccum* accession ‘MG5323’ (LatMg with 110 RILs) [54]; and one multi-parent population from the four-way cross combination of Neodur/Claudio//Colosseo/(Rascon/2*Tarro) with 338 lines (NCCR) [38].

Four *Pgt* races with different virulence phenotypes were tested on the plant populations in a greenhouse. Race TTTTF (isolate 02MN84A-1-2) is the most widely virulent race reported in the United States but also in Sicily (Italy), producing high ITs on all 20 of the wheat stem rust differential lines with the exception of *Sr24* and *Sr31* [4,55]. Race TPMKC (74MN1409) from the United States is virulent on *Sr36* [2]. Race JRCQC (isolate 09ETH80-3) is from Ethiopia and is virulent on both resistance genes *Sr9e* and *Sr13*, two genes constituting major components of stem rust resistance in durum cultivars and germplasm [7]. Race TKTTF (13ETH18-1), also from Ethiopia, is virulent on *SrTmp* [8]. The four races, with differential virulence specificities for many known stem rust resistance genes, were first used to evaluate the response of the parental lines. Then, races were individually used to score the specific mapping populations obtained by crossing parental genotypes with the resistant disease reactions of interest. Susceptible controls (cultivars ‘McNair 701′ and ‘Morocco’) were included in each experiment to monitor the infection level (density of uredinia on leaves) and virulence (maximum uredinial size) of the pathogen races. Additionally, 20 wheat stem rust differential lines were included in the experiments to confirm the identity and purity of *Pgt* races [4,55]. Resistant lines within the respective differential wheat sets served as the resistant controls, in addition to lines carrying *Sr24* (*LcSr24Ag*) and *Sr47* resistance gene.

### 4.2. Phenotypic Evaluation

Stem rust evaluations with foreign isolates were conducted in a Biosafety Level-3 Containment Facility on the St. Paul campus of the University of Minnesota (USA) during the winter seasons of 2016–2017 and 2019. Three seeds of each genotype and controls were sown in peat pot containers set within plastic 16 count trays, using a standard potting mix of 50% steam-treated native soil: 50% Metro mix (Sun Gro^®^ Horticulture, Agawam, MA, USA).

As described by Huang et al. [56], plants were grown in a rust-free greenhouse with a day/night temperature of 24/20 °C and 16-h photoperiod supplemented by 1000-watt high-pressure sodium (HPS) bulbs emitting 350–400 µmol·m^2^·s^−1^). Individual trays of plants were inoculated with 15 mg of rust suspended in 700 µL oil Soltrol 170 oil (Phillips Petroleum, Bartlesville, OK, USA) using an atomizing inoculator set at 0.035 MPa. After inoculation, plants were placed under lights (150–250 mmol photon/m^2^/s provided by 400 W sodium vapor lamps) for 2 h to hasten the evaporation of the oil carrier. Then, plants were pre-misted with household ultrasonic humidifiers continuously for 20 min before setting the misting regime to set for 2 min “on time” every 15 min for 16 h. After the initial 16 h infection period in the dark, lights were turned on for 2 h with the misters still running to complete the infection period. Thereafter, the misters were turned off and the plants were allowed to slow dry before moving back to the greenhouse.

ITs were assessed 12–14 days after inoculation using the 0–4 scale of Stakman et al. [57], where IT = 0 represents a highly resistant (immune) reaction and IT = 4 represents a susceptible (fully compatible) reaction. Raw IT data were converted to a 0–9 linear disease scale as follows: 0, 1−, 1, 1+, 2−, 2, 2+, 3−, 3, and 3+ were coded as 0, 1, 2, 3, 4, 5, 6, 7, 8, and 9, respectively, to carry out the genetic analysis. The semi-colon symbol used to represent a hypersensitive fleck “;” was converted to 0, while IT 4 was converted to 9 [58]. For lines with heterogeneous reactions, only the most prevalent IT was used. All the experiments were conducted in a completely randomized design with two replicates. Any accessions exhibiting variable reactions across the replicates were repeated in a second test.

### 4.3. Genetic Materials

A genetic map constructed with 398 DArT and PCR-based markers was available for the CirNeo population [53]. For the CicSve [38], LatMG [54] and NCCR [38] populations, genetic maps were previously developed with SNP markers from the Illumina wheat 90K iSelect array [59].

For all the maps, the marker segregation data were used for chi-square analyses to determine the segregation ratio and the deviation from the expected 1:1 ratio of each marker at *p* > 0.001. All markers with more than 10% missing data, and a null allele at one parent that was segregating for presence/absence in the mapping population, were excluded from further analysis. Physically mapped markers onto the reference durum genome [32] and marker data from the durum wheat consensus map [60] were used as anchor loci to compare the QTL map position across the four mapping populations.

### 4.4. Statistical and QTL Analyses

Frequency distributions of the phenotypic data were analyzed by chi-square (χ^2^) tests to estimate the complexity of genetic control of the traits and for alternative distributions. Toward this aim, disease reactions as given by IT values were used to categorize RILs into two classes (R for resistant, and S for susceptible) against each stem rust race. IT score 6 in the linear scale was selected as the upper threshold for resistance, since it corresponds to small-to medium-sized uredinia often surrounded by chlorosis or necrosis, a phenotype indicative of some resistance. Analysis of variance (ANOVA) of IT scoring values was performed to test the significance of differences between RILs and replications using the software Statistica 7 (Statsoft Europe, Hamburg, Germany).

For biparental populations, QTL detection was performed in QGene 8.3.16 using composite interval mapping [61]. A scanning interval of 2 cM between markers and putative QTLs with a window size of 10 cM was used to detect QTLs. The marker cofactors for background control were set by single marker regression and simple interval analysis, with a maximum of five controlling markers. Putative QTLs were defined as two or more linked markers that were associated with a trait at a log10 odds ratio (LOD) ≥ 3. Suggestive QTLs at the subthreshold of 2.0 < LOD < 3.0 were also reported as targets for future investigations [62]. The additive effects of QTLs were estimated as half of the difference between the phenotypic values of the respective homozygotes. The 95% confidence intervals (CIs) for identified QTL in their original maps were calculated through the LOD-2 criterion.

For the NCCR population, a haplotype-based QTL analysis was performed. Firstly, the intervals of the haplotype blocks were determined using the “LD spine” algorithm implemented within the “Haploview” 4.2 software (Broad Institute, Cambridge, MA, USA) [63], setting a value of d’ = 0.8. Subsequently, the haplotype form call was performed for all blocks for all lines in the population and the adherence of the observed haplotype frequencies to the expected ones was ascertained. The phenotype/haplotype association analysis was performed using a two-step procedure: initially, a single haplotype block analysis was performed by ANOVA using the phenotype as the dependent variable and the haplotype forms at each individual block as the independent variable. In the second step, haplotype blocks that were significantly associated (*p* value < 0.0001) were subjected to forward stepwise regression using the Bayesian Information Criterion for the retention or rejection of haplotype blocks in the global model. The procedure was repeated using the previously identified haplotype blocks as covariates in the single haplotype block analysis and subsequent stepwise regression. Once the most associated haplotype blocks were identified, tag-markers and confidence intervals were determined; the tag-marker of a haplotype block was defined as the marker most associated with the phenotype among the markers of the same haplotype block. The confidence interval was determined on the basis of the position of the most distant markers to the left and right of the tag-marker for which a value of *r^2^* > 0.5 was observed with the tag marker itself. Statistical analysis was conducted in R using the car package [64,65].

The QTLs were projected onto the durum wheat consensus map by Maccaferri et al. [32] and on Svevo genome. The allelic state for SNP markers underlying QTLs on chromosome 6A for the parents of the segregating populations has been retrieved from the Global Durum Genomic Resource (https://wheat.pw.usda.gov/GG3/global_durum_genomic_resources, accessed on 1 June 2022) and unpublished data.

### 4.5. Identification of Candidate Genes

Putative candidate genes were identified for the detected QTL/MQTL for stem rust resistance. For each QTL, peak markers and flanking markers corresponding to the CIs were located on the reference Svevo genome based on the BLAST matches (BLASTN, E-value = E^−10^) of the nucleotide sequences of the corresponding SNP and DArT markers [32,53,59]. For the CirNeo population, intervals larger than the calculated CIs were considered (10 Mbp) as very few markers were mapped to the linkage groups in which QTLs were identified. The physical region underpinned by QTLs was inspected to retrieve all the genes with their functional annotations and to identify candidate genes for *Pgt* resistance.

## 5. Conclusions

In conclusion, the present study provides a collection of loci for stem rust resistance in tetraploid wheat segregating populations. The use of the 90K SNPs for most populations allowed us to compare our results with those of similar studies, and provided a set of molecular markers to simultaneously select multiple beneficial alleles for disease resistance in durum wheat breeding programs. The availability of the genome sequence in durum wheat is of paramount importance to identify high-quality haplotypes for the correct association of molecular markers with phenotypes and to identify candidate genes for resistance loci.

## Figures and Tables

**Table 1:** Chi-square tests for segregation of stem rust reaction in four recombinant inbred line populations of durum wheat.

RIL Population Name	Stem Rust Race	No. of Resistant RILs (IT ≤ 6)	No. of Susceptible RILs (IT > 6)	Observed Segregation	χ^2^	No. of Expected Genes
LatMG	TPKMC	66	47	1:1	(1, N = 113) = 3.2, *p* > 0.10	1 gene
LatMG	JRCQC	82	27	3:1	(1, N = 109) = 0.003, *p* > 0.90	2 genes, both from MG5323
LatMG	TKTTF	85	25	3:1	(1, N = 110) = 0.30, *p* > 0.50	2 genes, both from MG5323
CicSve	TPMKC	36	38	1:1	(1, N = 74) = 0.054, *p* > 0.80	1 gene
CicSve	TKTTF	25	25	1:1	exact segregation	1 gene
CirNeo	TTTTF	85	26	3:1	(1, N = 111) = 0.147, *p* > 0.50	2 genes, one per parent
NCCR	TTTTF	213	122	5:3	(1, N = 335) = 0.167, *p* > 0.50	3 genes: one independent by two parents, and two dependent on one parent

**Table 2:** Statistic parameters for reaction of the mapping populations to four races of *Puccinia graminis* f. sp. *tritici*. CV: coefficient of variation; MSD: minimum significant difference; H^2^: heritability.

Race	Population	Mean	Range	CV	MSD	Genetic Variance	H^2^
TPMKC	LatMG	4.33	0.0–9.0	0.81	2.00	6.76	0.81
JRCQC	LatMG	3.16	0.0–9.0	0.98	1.40	5.75	0.89
TKTTF	LatMG	3.11	0.0–9.0	0.96	2.11	4.69	0.74
TPMKC	CicSve	5.21	0.0–9.0	0.68	1.15	5.63	0.92
TKTTF	CicSve	5.44	3.0–9.0	0.59	2.00	3.96	0.73
TTTTF	CirNeo	2.34	0.0–9.0	1.53	0.76	8.23	0.98
TTTTF	NCCR	3.78	0.0–9.0	0.94	0.27	8.35	0.99

**Table 3:** Correlation analysis between reaction profile of: (a) LatMG RILs against the three considered races: TPMKC, JRCQC, and TKTTF, and (b) CicSve RILS against the two races TPMKC and TFTTF. Pearson correlation R values are reported. All reported correlation values are significant at *p* < 0.01.

(a)				
LatMG		JRCQC		TKTTF
**TPMKC**		0.646		0.598
**JRCQC**				0.893
**(b)**				
**CicSve**			**TKTTF**	
**TPMKC**			0.946	

**Table 4:** QTLs identified in the present study. Add. Effect: additive effect; CI: confidence interval; Left and Right pos. are physical position of left and right marker on the durum wheat reference genome of Svevo. * For QSr_CxN.6A.2 the physical position of the left end was estimated based on other markers present in the region, as the Ug99-6A marker was a phenotypic marker related to resistance to race TTKSK [34].

QTL Name	Trait	Population	Peak Marker	Linkage Group	Position (cM)	LOD	R^2^	Add. Effect	CI Start (cM)	CI End (cM)	Left Marker	Right Marker	Left Pos.	Right Pos.	Resistant Parent
QSr_CxS.3A.1	TKTTF	CicSve	IWB66101	3A_2	6	3.5	0.28	1.74	3.6	8.4	IWB73868	IWB71206	97,532,661	151,028,408	Svevo
QSr_CxS.3A.2	TPMKC	CicSve	IWB66101	3A_2	6	2.2	0.13	1.28	0.8	11.2	IWB61873	IWB71206	66,269,458	151,028,408	Svevo
QSr_LxM.4A.1	JRCQC	LatMG	IWB72220	4A	152.9	15.9	0.35	1.55	143.2	153.5	IWB9431	IWA2756	715,353,110	721,414,881	MG5323
QSr_LxM.4A.2	TKTTF	LatMG	IWB72220	4A	152.9	17.1	0.42	1.56	143.2	153.5	IWB9431	IWA2756	715,353,110	721,414,881	MG5323
QSr_NCCR.6A.1	TTTTF	NCCR	IWB48751	6A	7	39.1	0.27	4.39	0	7	IWB40670	IWB48751	1,202,560	7,425,521	Neodur
QSr_CxN.6A.1	TTTTF	CirNeo	Xgwm459	6A-1	95.8	13.3	0.42	2.286	94.2	95.8	Xgwm459	Xgwm1040	6,193,023	27,620,384	Neodur
QSr_NCCR.6A.2	TTTTF	NCCR	IWB29924	6A	116.7	54.9	0.36	4.4	10.7	116.7	IWB71246	IWB29924	591,646,505	602,662,186	Claudio-Rascon/2*Tarro
QSr_NCCR.6A.3	TTTTF	NCCR	IWB60184	6A	125.7	70.9	0.46	4.81	125.7	125.7	IWB24237	IWB44475	613,056,324	613,257,397	Claudio-Rascon/2*Tarro
QSr_CxS.6A.1	TKTTF	CicSve	IWB69393	6A_5	4	27.9	0.92	2.99	3.3	4.7	IWB73686	IWB69393	607,669,872	611,710,729	Svevo
QSr_CxS.6A.2	TPMKC	CicSve	IWB69393	6A_5	4	40.4	0.92	3.399	3.3	4.7	IWB73686	IWB69393	607,669,872	611,710,729	Svevo
QSr_CxN.6A.2	TTTTF	CirNeo	Ug99-6A	6A-2	0	9.9	0.34	1.763	0	4	Xgwm169	Ug99-6A *	592,907,113	614,545,144	Cirillo
QSr_LxM.6B.1	TPKMC	LatMG	IWB60699	6B	148.7	23.3	0.68	2.91	147.6	148.7	IWB8910	IWB60699	690,927,250	695,093,664	MG5323
QSr_LxM.6B.2	JRCQC	LatMG	IWB60699	6B	148.7	18.5	0.44	1.77	147	148.7	Xwmc621a	IWB60699	685,341,777	695,093,664	MG5323
QSr_LxM.6B.3	TKTTF	LatMG	IWB58435	6B	148.1	16.9	0.41	1.46	146.3	148.7	Xwmc621a	IWB60699	685,341,777	695,093,664	MG5323

**Table 5:** Size and gene content of the physical regions corresponding to the QTLs for stem rust resistance identified in the present study.

QTL	Interval Svevo (Mbp)	Number of Annotated Genes	Ratio Annotated Genes/Mbp	Number of Disease-Related Genes	Ratio Disease-Related/Annotated Genes	Ratio Disease-Related Genes/Mbp
QSr_CxS.3A.1	53.5	319	6.0	33	0.1	0.6
QSr_CxS.3A.2	84.8	517	6.1	47	0.1	0.5
QSr_LxM.4A.1/2	6.1	139	23.3	20	0.1	3.3
QSr_NCCR.6A.1	6.2	134	21.5	22	0.2	3.5
QSr_CxN.6A.1	21.4	927	43.3	116	0.1	5.4
QSr_NCCR.6A.2	11.0	712	64.6	46	0.1	4.2
QSr_NCCR.6A.3	0.2	18	89.5	2	0.1	10.0
QSr_CxS.6A.1/2	4.0	46	11.4	11	0.2	2.7
QSr_CxN.6A.2	21.6	1136	52.5	116	0.1	5.4
QSr_LxM.6B.1	4.2	210	50.4	30	0.1	7.1
QSr_LxM.6B.2/3	9.8	422	43.3	72	0.2	7.3

## Supplementary Materials

The following supporting information can be downloaded at: https://www.mdpi.com/article/10.3390/genes13101793/s1, Table S1: Allelic state for the markers comprised in the region of QTLs on chromosome 6A for the parents of the segregating populations and additional genotypes carrying different alleles at the *Sr13* locus; Table S2: List of annotated genes in physical intervals of QTLs identified in the present study; Table S3: Reaction to *Pgt* races of parents of the segregating populations and differential lines.
